# Supplementary material for: A new diatom-based multimetric index to assess lake ecological status
Source: Environ Monit Assess. 2023 Sep 13;195(10):1202. doi: 10.1007/s10661-023-11855-w (PMC10499699; doi:10.1007/s10661-023-11855-w)
Supplement: Supplementary file 1 — Supplementary file1 (RTF 2019 KB) [file 10661_2023_11855_MOESM1_ESM.rtf]

			Alert taxa (1: yes ; O: no)	
code	name	author	BOD5	SP	NKJ	Pt	
AAMB	Aulacoseira ambigua	(Grunow) Simonsen	1	0	1	1	
ABRT	Achnanthidium bioretii	(Germain) Edlund	0	0	0	0	
ABRT	Achnanthidium bioretii	(Germain) Monnier Lange-Bertalot & Ector	0	0	0	0	
ABRY	Adlafia bryophila	(Petersen) Lange-Bertalot in Moser & al.	0	0	0	0	
ACAF	Achnanthidium affine	(Grun) Czarnecki	0	0	0	0	
ACLI	Achnanthidium lineare	W. Smith	0	0	0	0	
ACOP	Amphora copulata	(Kützing) Schoeman & Archibald	1	1	1	0	
ADAM	Achnanthidium atomoides	Monnier, Lange-Bertalot & Ector	0	0	0	0	
ADAS	Achnanthidium anastasiae	(Kaczmarska) Chudaev et Gololobova	0	0	0	0	
ADCA	Achnanthidium caledonicum	Lange-Bertalot)Lange-Bertalot	0	0	0	0	
ADCT	Achnanthidium catenatum	(Bily & Marvan) Lange-Bertalot	0	0	0	1	
ADDA	Achnanthidium daonense	(Lange-Bertalot) Lange-Bertalot Monnier & Ector	0	0	0	0	
ADEG	Achnanthidium exiguum	(Grunow) Czarnecki	1	0	0	1	
ADEU	Achnanthidium eutrophilum	(Lange-Bertalot)Lange-Bertalot	1	1	0	0	
ADEX	Achnanthidium exile	(Kützing) Heiberg	0	0	0	0	
ADEX	Achnanthidium exile	(Kützing) Bukhtiyarova	0	0	0	0	
ADEX	Achnanthidium exile	(Kützing) Round & Bukhtiyarova	0	0	0	0	
ADGL	Achnanthidium gracillimum	(Meister)Lange-Bertalot	0	0	0	0	
ADHE	Achnanthidium helveticum	(Hustedt) Monnier Lange-Bertalot & Ector	0	0	0	0	
ADJK	Achnanthidium jackii	Rabenhorst	0	0	0	0	
ADKR	Achnanthidium kranzii	(Lange-Bertalot) Round & Bukhtiyarova	0	0	0	0	
ADLA	Achnanthidium latecephalum	Kobayasi	0	0	0	0	
ADMC	Achnanthidium microcephalum	Kützing sensu W. Smith	0	0	0	0	
ADMI	Achnanthidium minutissimum	(Kützing) Czarnecki group 1	0	0	0	0	
ADMI	Achnanthidium minutissimum	(Kützing) Czarnecki group 2	0	0	0	0	
ADMI	Achnanthidium minutissimum	(Kützing) Czarnecki group 3	0	0	0	0	
ADMI	Achnanthidium minutissimum	(Kützing) Czarnecki	0	0	0	0	
ADMO	Achnanthidium delmontii	PeRes. Le Cohu et Barthes	0	0	0	0	
ADMS	Adlafia minuscula	(Grunow) Lange-Bertalot	0	0	0	0	
ADMU	Adlafia muralis	(Grunow in Van Heurck 1880) Li et Qi	0	0	0	0	
ADMU	Adlafia muralis	(Grunow) Monnier & Ector	0	0	0	0	
ADNM	Achnanthidium neomicrocephalum	Lange-Bertalot & Staab	0	0	0	0	
ADPL	Achnanthidium pseudolineare	Van de Vijver. Novais et Ector	0	0	0	0	
ADPS	Achnanthidium petersenii	(Hustedt) C.E. Wetzel,Ector, D.M. Williams & Jüttner	0	0	0	0	
ADPY	Achnanthidium pyrenaicum	(Hustedt) Kobayasi	0	0	0	0	
ADRI	Achnanthidium rivulare	Potapova & Ponader	0	0	0	1	
ADRK	Achnanthidium rosenstockii	(Lange-Bertalot) Lange-Bertalot in Krammer & Lange-Bertalot	0	0	0	0	
ADRU	Achnanthidium druartii	Rimet & Couté in Rimet & al.	0	1	0	0	
ADSA	Achnanthidium saprophilum	(Kobayasi et Mayama) Round & Bukhtiyarova	0	0	0	0	
ADSB	Achnanthidium straubianum	(Lange-Bertalot)Lange-Bertalot	0	0	0	0	
ADSH	Achnanthidium subhudsonis	(Hustedt) H. Kobayasi	0	0	0	0	
ADSO	Achnanthidium subatomoides	(Hustedt) Monnier, Lange-Bertalot et Ector	0	0	0	0	
ADSU	Achnanthidium subatomus	(Hustedt) Lange-Bertalot	0	0	0	0	
ADTC	Achnanthidium tropicocatenatum	Marquardt, C.E.Wetzel & Ector	0	0	0	0	
ADTR	Achnanthidium trinode	Ralfs in Pritchard	0	0	0	0	
AFOR	Asterionella formosa	Hassall	0	0	0	0	
AFOR	Asterionella formosa	Hassall	0	0	0	0	
AGRU	Achnanthes grubei	Simonsen	0	0	0	0	
AGSL	Aulacoseira granulata	(Ehrenberg) Simonsen	1	0	1	1	
AGSL	Aulacoseira granulata	(Ehrenberg) Simonsen	1	0	1	1	
AHOF	Achnanthidium hoffmannii	Van de Vijver. Ector, Mertens & Jarlman	0	0	0	0	
AINA	Amphora inariensis	Krammer	0	0	0	0	
ALBL	Adlafia langebertalotii	Monnier et Ector	0	0	0	0	
AMCD	Amphora macedoniensis	Nagumo	0	0	0	0	
AMDN	Amphora meridionalis	Levkov	0	0	0	0	
AMID	Amphora indistincta	Levkov	0	0	0	0	
AMLB	Amphora lange-bertalotii	Levkov, & Metzeltin	1	1	1	1	
AMUZ	Aulacoseira muzzanensis	(Meister) Krammer	1	1	1	1	
ANMN*	Actinocyclus normanii morphotype normanii	(Gregory ex Greville) Hustedt	0	0	0	0	
ANRS	Aneumastus rosettae	Lange-Bertalot & Miho	0	0	0	0	
ANSS	Aneumastus stroesei	(Østrup) Mann & Stickle in Round Crawford & Mann	0	0	0	0	
AOVA*	Amphora ovalis var. ovalis	(Kützing) Kützing	0	0	0	0	
APED	Amphora pediculus	(Kützing) Grunow	0	1	0	1	
APEL	Amphipleura pellucida	Kützing	0	0	0	0	
APFI	Achnanthidium pfisteri	Lange-Bertalot	0	0	0	0	
AUAL	Aulacoseira alpigena	Grunow) Krammer	0	0	0	0	
AUGR	Aulacoseira granulata	(Ehrenberg) Simonsen	1	1	1	1	
AUGR	Aulacoseira granulata	(Ehrenberg) Simonsen	1	1	1	1	
AUPU	Aulacoseira pusilla	(Meister) Tuji et Houki	1	1	1	1	
AUSL	Aulacoseira scalaris	(Grunow) Houk, Klee & Passauer	0	0	0	0	
AUSU	Aulacoseira subarctica	(O. Müller) Haworth	0	0	0	0	
AUTL	Aulacoseira tenella	(Nygaard) Simonsen	0	0	0	0	
AUVA	Aulacoseira valida	Grunow)Krammer	0	1	1	1	
AVTU	Amphora vetula	Levkov,	0	0	0	0	
AZHA	Achnanthidium zhakovschikovii	M. Potapova	0	0	0	0	
BBRE*	Brachysira brebissonii subsp. brebissonii	Ross in Hartley	0	0	0	0	
BGAR	Brachysira garrensis	(Lange-Bertalot & Krammer) Lange-Bertalot	0	0	0	0	
BLIL	Brachysira liliana	Lange-Bertalot	0	0	0	0	
BMIC	Brachysira microcephala	(Grunow) Compère	0	0	0	0	
BNEG	Brachysira neglectissima	Lange-Bertalot	0	0	0	0	
BNEO	Brachysira neoexilis	Lange-Bertalot	0	0	0	0	
BPAX	Bacillaria paxillifera	(O.F. Müller) Hendey	0	0	0	0	
BPRO	Brachysira procera	Lange-Bertalot & Moser	0	0	0	0	
BVIT	Brachysira vitrea	(Grunow) Ross in Hartley	0	0	0	0	
CAEX*	Cymbella excisa var. excisa	Kützing	0	1	0	0	
CAFF*	Cymbella affinis var. affinis	Kützing	0	0	0	0	
CAFM	Cymbella affiniformis	Krammer	0	0	0	0	
CAMB	Craticula ambigua	(Ehrenberg) Mann	0	0	0	0	
CAPS	Caloneis alpestris	(Grunow) Cleve	0	0	0	0	
CATE	Caloneis tenuis	(Gregory) Krammer	0	0	0	0	
CATO	Cyclotella atomus	Hustedt	0	1	1	1	
CBAC	Caloneis bacillum	(Grunow) Cleve	0	0	0	0	
CBAM	Cymbopleura amphicephala	Krammer	0	0	0	0	
CBHD	Cymbopleura hustedtii	Novelo Tavera & Ibarra	0	0	0	0	
CBKU*	Cymbopleura kuelbsii var. kuelbsii	Krammer	0	0	0	0	
CBNA*	Cymbopleura naviculiformis var. naviculiformis	(Auerswald) Krammer	0	0	0	0	
CBPY	Cymbopleura pyrenaica	Le Cohu et Lange-Bertalot	0	0	0	0	
CCMP	Cymbella compacta	Østrup	0	0	0	0	
CCOC	Cavinula cocconeiformis	(Gregory ex Greville) Mann & Stickle in Round Crawford & Mann	0	0	0	0	
CCYM	Cymbella cymbiformis	Agardh	0	0	0	0	
CDTG*	Cyclotella distinguenda var. distinguenda	Hustedt	0	0	0	0	
CDUB	Cyclostephanos dubius	(Fricke) Round	1	1	1	0	
CEUG	Cocconeis euglypta	Ehrenberg	1	1	1	0	
CEXF*	Cymbella excisiformis var. excisiformis	Krammer	0	0	0	0	
CFDI	Cymbellafalsa diluviana	(Krasske) Lange-Bertalot & Metzeltin	0	0	0	0	
CFON	Caloneis fontinalis	(Grunow) Lange-Bertalot & Reichardt	1	1	1	1	
CFON	Caloneis fontinalis	(Grunow in Van Heurck) Cleve-Euler	1	1	1	1	
CFTF	Cymbopleura florentiniformis	Krammer	0	0	0	0	
CHEL	Cymbella helvetica	Kützing	0	0	0	0	
CHHA	Chamaepinnularia hassiaca	(Krasske) Cantonati & Lange-Bertalot	0	0	0	0	
CHLI	Craticula halophilioides	(Hustedt) Lange-Bertalot	0	0	0	0	
CHME	Chamaepinnularia mediocris	(Krasske) Lange-Bertalot in Lange-Bertalot & Metzeltin	0	0	0	0	
CINV	Cyclostephanos invisitatus	Hohn & Hellerman)Theriot Stoermer & Håkansson	0	0	0	0	
CJAR	Cavinula jaernefeltii	(Hustedt) Mann & Stickle in Round Crawford & Mann	0	0	0	0	
CKPP	Cymbella kappii	(Cholnoky) Cholnoky	0	0	0	0	
CLAE*	Cymbella laevis var. laevis	Naegeli ex Kützing	0	0	0	0	
CLBE	Cymbella lange-bertalotii	Krammer	0	0	0	0	
CLCT	Caloneis lancettula	(Schulz) Lange-Bertalot & Witkowski	1	1	1	1	
CLNT	Cocconeis lineata	Ehrenberg	1	0	0	1	
CLTL	Cymbella lancettula	(Krammer) Krammer	0	0	0	0	
CMDU	Cyclotella meduanae	Germain emend Genkal	1	1	1	1	
CMDU	Cyclotella meduanae	Germain	1	1	1	1	
CMEN	Cyclotella meneghiniana	Kützing	1	1	1	1	
CMLF	Craticula molestiformis	(Hustedt) Lange-Bertalot	0	0	0	0	
CNCI*	Cymbella neocistula var. neocistula	Krammer	0	0	0	0	
CNLC	Cymbella neolanceolata	W. Silva	0	0	0	0	
CNLP*	Cymbella neoleptoceros var. neoleptoceros	Krammer	0	1	0	1	
CNTH	Cocconeis neothumensis	Krammer	0	0	0	0	
COPL	Cocconeis pseudolineata	(Geitler) Lange-Bertalot	0	0	0	0	
CPAR	Cymbella parva	(W. Sm.) Kirchner in Cohn	0	0	0	0	
CPED	Cocconeis pediculus	Ehrenberg	0	0	0	0	
CPLA*	Cocconeis placentula var. placentula	Ehrenberg	0	0	0	0	
CPPV	Cymbella perparva	Krammer	0	0	0	0	
CPRX*	Cymbella proxima var. proxima	Reimer in Patrick & Reimer	0	0	0	0	
CPSE	Cavinula pseudoscutiformis	(Hustedt) Mann & Stickle in Round Crawford & Mann	0	0	0	0	
CRAC	Craticula accomoda	(Hustedt) Mann	0	0	0	0	
CRBU	Craticula buderi	(Hustedt) Lange-Bertalot	0	0	0	0	
CRCU	Craticula cuspidata	(Kützing) Mann	0	0	0	0	
CSAQ*	Cymbopleura subaequalis var. subaequalis	(Grunow) Krammer	0	0	0	0	
CSBH	Cymbella subhelvetica	Krammer	0	0	0	0	
CSCI	Cymbella subcistula	Krammer	0	0	0	0	
CSDL	Cyclostephanos delicatus	(Genkal) Kling & Håkansson	0	0	0	0	
CSDL	Cyclostephanos delicatus	(Genkal) Casper & Scheffler	0	0	0	0	
CSHU	Caloneis schumanniana	(Grunow in Van Heurck) Cleve	0	0	0	0	
CSIL	Caloneis silicula	(Ehrenberg) Cleve	0	0	0	0	
CSLP	Cymbella subleptoceros	Krammer	1	0	0	1	
CSMU	Chamaepinnularia submuscicola	(Krasske) Lange-Bertalot	0	0	0	0	
CSNU	Craticula subminuscula	(Manguin) C.E. Wetzel & Ector	1	1	1	1	
CSUT*	Cymbella subtruncata var. subtruncata	Krammer	0	0	0	0	
CTPU	Ctenophora pulchella	(Ralfs ex Kütz.) Williams et Round	0	0	0	0	
CTRQ	Centric diatoms	Diatomées centriques indifférenciées	0	0	0	0	
CTUM	Cymbella tumida	(Brébisson)Van Heurck	1	1	0	1	
CVMO	Cavinula mollicula	(Hustedt) Lange-Bertalot	0	0	0	0	
CVSO	Cavinula scutelloides	(W. Smith) Lange-Bertalot	0	0	0	0	
CVUL*	Cymbella vulgata var. vulgata	Krammer	0	0	0	0	
DCAL	Diploneis calcilacustris	Lange–Bertalot et A. Fuhrmann	0	0	0	0	
DCOF*	Diadesmis confervacea var. confervacea	Kützing	0	1	1	1	
DEFO*	Diatomée anormale f. anormale	Abnormal diatom valve (unidentified) or sum of deformities abundance	0	1	1	1	
DEHR	Diatoma ehrenbergii	Kützing	0	0	0	0	
DITE	Diatoma tenue	Agardh	1	0	0	1	
DKOT	Dorofeyukea kotschyi	(Grunow) Kulikovskiy, Kociolek, Tusset & T.Ludwig	0	0	0	0	
DKRA	Diploneis krammeri	Lange-Bertalot & Reichardt	0	0	0	0	
DKUE*	Denticula kuetzingii var. kuetzingii	Grunow	0	0	0	0	
DMES	Diatoma mesodon	(Ehrenberg) Kützing	0	0	0	0	
DOBL	Diploneis oblongella	(Naegeli) Cleve-Euler	0	0	0	0	
DOCU	Diploneis oculata	(Brébisson in Desmazières) Cleve	0	0	0	0	
DPAR	Diploneis parma	Cleve	0	0	0	0	
DPDE	Delicatophycus delicatulus	(Kützing) M.J.Wynne .	0	0	0	0	
DPSG	Discostella pseudostelligera	(Hustedt) Houk & Klee emend. Genkal	0	0	0	1	
DPSG	Discostella pseudostelligera	(Hustedt) Houk et Klee	0	0	0	1	
DSEP	Diploneis separanda	Lange-Bertalot	0	0	0	0	
DSTE	Discostella stelligera	(Cleve et Grun.) Houk & Klee	0	0	0	0	
DTEN	Denticula tenuis	Kützing	0	0	0	0	
DVUL	Diatoma vulgaris	Bory	0	0	0	0	
EADN	Epithemia adnata	(Kützing) Brébisson	1	0	1	1	
EARB	Eunotia arcubus	Nörpel-Schempp & Lange-Bertalot	0	0	0	0	
EARC*	Eunotia arcus var. arcus sensu stricto	Ehrenberg	0	0	0	0	
EAUE	Encyonema auerswaldii	Rabenhorst	0	0	0	0	
EBLU	Eunotia bilunaris	(Ehrenberg) M.G.M. Souza in Souza & Moreira-Filho	0	0	0	0	
EBLU	Eunotia bilunaris	(Ehrenberg) Schaarschmidt	0	0	0	0	
EBNA	Encyonema bonapartei	HeudrE. C.E. Wetzel & Ector	0	0	0	0	
EBOA	Eunotia boreoalpina	Lange-Bertalot & Nörpel-Schempp	0	0	0	0	
EBOT	Eunotia botuliformis	Wild, Nörpel-Schempp & Lange-Bertalot	0	0	0	0	
EBOT	Eunotia botuliformis	Wang	0	0	0	0	
ECAE*	Encyonema caespitosum var. caespitosum	Kützing	0	0	0	0	
ECAL	Encyonopsis alpina	Krammer & Lange-Bertalot	0	0	0	0	
ECES	Encyonopsis cesatii	(Rabenhorst) Krammer	0	0	0	0	
ECKR	Encyonopsis krammeri	Reichardt	0	0	0	0	
ECPM	Encyonopsis minuta	Krammer & Reichardt	0	0	0	0	
ECTA	Encyonopsis tavirana	Krammer	0	0	0	0	
EEXI	Eunotia exigua	(Brébisson ex Kützing) Rabenhorst	0	0	0	0	
EFAB	Eunotia faba	(Ehrenberg) Grunow in Van Heurck	0	0	0	0	
EGBA	Epithemia gibba	(Ehrenberg) Kützing	1	0	1	1	
EHOR	Encyonopsis horticola	Van de Vijver, Lange-Bertalot & Compère	0	0	0	0	
EIMP	Eunotia implicata	Nörpel Lange-Bertalot & Alles	0	0	0	0	
EINC*	Eunotia incisa var. incisa	Gregory	0	0	0	0	
ELBV*	Encyonema lange-bertalotii var. lange-bertalotii	Krammer	0	0	0	0	
ELEI	Encyonema leibleinii	(C. Agardh) Silva, Jahn Ludwig & Menezes	0	1	0	0	
EMIN	Eunotia minor	(Kützing) Grunow in Van Heurck	0	0	0	0	
EMIN	Eunotia minor	Fusey	0	0	0	0	
EMUC	Eunotia mucophila	(Lange-Bert.&Norpel Schempp) Lange-Bertalot	0	0	0	0	
ENAE	Eunotia naegelii	Migula	0	0	0	0	
ENCM	Encyonopsis microcephala	(Grunow) Krammer	0	0	0	0	
ENEE	Encyonopsis neerlandica	Van de Vijver. Verweij, Van Der Wal & Mertens	0	0	0	0	
ENKA	Encyonema kalbei	Krammer	0	0	0	0	
ENMI	Encyonema minutum	(Hilse in Rabh.) D.G. Mann in Round Crawford & Mann	0	1	1	1	
ENNG	Encyonema neogracile	Krammer	0	0	0	0	
ENRE	Encyonema reichardtii	(Krammer) D.G. Mann in Round Crawford & Mann	0	0	0	0	
ENRO	Encyonema rostratum	Krammer	0	0	0	0	
ENTR	Encyonema triangulum	(Ehrenberg) Kützing	0	1	0	0	
ENVE	Encyonema ventricosum	(Kützing) Grunow in Schmidt & al.	0	0	0	0	
EOCO	Eolimna comperei	Ector Coste et Iserentant in Coste & Ector	0	0	0	0	
EORT	Eunotia orthohedra	Furey, Lowe et Johansen	0	0	0	0	
EPBO	Epithemia proboscidea	Kützing	0	0	0	0	
EPEC*	Eunotia pectinalis var. pectinalis	(Kützing) Rabenhorst	0	0	0	0	
EPHP	Epithemia parallela	(Grunow) Ruck & Nakov	0	0	0	0	
EPHP	Epithemia parallela	Proschkina-Lavrenko	0	0	0	0	
EREI	Epithemia reicheltii	Fricke	0	0	0	0	
ERHO	Eunotia rhomboidea	Hustedt	0	0	0	0	
ESLE	Encyonema silesiacum	(Bleisch in Rabh.) D.G. Mann	0	1	0	1	
ESMI	Epithemia smithii	Carruthers in Gray	0	0	0	0	
ESOR	Epithemia sorex	Kützing	1	1	1	1	
ESUB	Eunotia subarcuatoides	Alles Nörpel & Lange-Bertalot in Alles et al.	0	0	0	0	
ESUM	Encyonopsis subminuta	Krammer & Reichardt	0	0	0	0	
ETEN	Eunotia tenella	(Grunow in Van Heurck) Hustedt in Schmidt & al	0	0	0	0	
ETUR*	Epithemia turgida var. turgida	(Ehrenberg) Kützing	0	0	0	0	
EUAL	Eucocconeis alpestris	(Brun) Lange-Bertalot	0	0	0	0	
EUBI	Eunotia bidens	Ehrenberg	0	0	0	0	
EUFL	Eucocconeis flexella	(Kützing) Meister	0	0	0	0	
EULA	Eucocconeis laevis	(Østrup) Lange-Bertalot	0	0	0	0	
EVUL*	Encyonema vulgare var. vulgare	Krammer	0	1	0	0	
FAPO	Fragilaria amphicephaloides	Lange-Bertalot in Hofmann & al.	0	0	0	0	
FAQU	Fragilaria aquaplus	Lange-Bertalot & Ulrich	0	0	0	0	
FAUT	Fragilaria austriaca	(Grunow) Lange-Bertalot	0	0	0	0	
FCRO	Fragilaria crotonensis	Kitton	0	0	0	0	
FCRS	Frustulia crassinervia	(Breb.) Lange-Bertalot et Krammer	0	0	0	0	
FERI	Frustulia erifuga	Lange-Bertalot & Krammer	0	0	0	0	
FFBI	Fragilariforma bicapitata	(A.Mayer) Williams & Round	0	0	0	0	
FFNI	Fragilariforma nitzschioides	(Grunow) Lange-Bertalot in Hofmann Werum & Lange-Bertalot	0	0	0	0	
FFUN	Fragilariforma undata	(W.Smith) Heudre, C.E.Wetzel & Ector	0	0	0	0	
FFUS	Fragilaria fusa	(R.M. Patrick) Wengrat, C.E. Wetzel & E. Morales	1	1	0	1	
FFVI	Fragilariforma virescens	(Ralfs) Williams & Round	0	0	0	0	
FGRA	Fragilaria gracilis	Østrup	0	0	0	0	
FLEN	Fallacia lenzii	Hustedt) Lange-Bertalot	0	0	0	0	
FLEN	Fallacia lenzii	(Hustedt) Mann in Van de Vijver & al	0	0	0	0	
FMES	Fragilaria mesolepta	Rabenhorst	0	0	0	0	
FMIT	Fallacia mitis	(Hustedt) D.G. Mann	0	0	0	0	
FMIV	Fragilaria microvaucheriae	C.E. Wetzel et Ector	0	0	0	0	
FNEV	Fragilaria nevadensis	Linares-Cuesta & Sanchez-Castillo	0	0	0	0	
FNIN	Fragilaria neointermedia	Tuji et D.M. Williams	0	0	0	0	
FPDE	Fragilaria perdelicatissima	Lange-Bertalot & Van de Vijver	0	0	0	0	
FPEC	Fragilaria pectinalis	Lyngbye	0	1	0	1	
FPEM	Fragilaria perminuta	(Grunow) Lange-Bertalot	0	0	0	0	
FPRU	Fragilaria pararumpens	Lange-Bertalot, Hofmann & Werum in Hofmann & al.	0	1	1	1	
FRAD	Fragilaria radians	(Kütz.) Williams & Round	0	0	0	0	
FRAD	Fragilaria radians	Lange-Bertalot in Hofmann & al.	0	0	0	0	
FRUM	Fragilaria rumpens	(Kütz.) G.W.F. Carlson	0	1	0	1	
FSAP	Fistulifera saprophila	(Lange-Bertalot & Bonik) Lange-Bertalot	0	0	0	0	
FSAX	Frustulia saxonica	Rabenhorst	0	0	0	0	
FSBH	Fallacia subhamulata	(Grunow in V. Heurck) D.G. Mann	0	0	0	0	
FSCS	Fragilaria subconstricta	Østrup	0	0	0	0	
FSCS	Fragilaria subconstricta	Østrup (Oestrup 1910) emend Heudre	0	0	0	0	
FSLU	Fallacia sublucidula	(Hustedt) D.G. Mann	0	0	0	0	
FSOC	Fragilaria socia	(Wallace) Lange-Bertalot	0	0	0	0	
FSXP	Fragilaria saxoplanctonica	Lange-Bertalot & Ulrich	0	0	0	0	
FTEN	Fragilaria tenera	(W. Smith) Lange-Bertalot	0	0	0	0	
FTNU	Fragilaria tenuissima	Lange-Bertalot & Ulrich	0	0	0	0	
FVAU*	Fragilaria vaucheriae var. vaucheriae	(Kützing) Petersen	1	1	1	1	
FVUL	Frustulia vulgaris	(Thwaites) De Toni	0	0	0	0	
GACC	Geissleria acceptata	(Hustedt) Lange-Bertalot & Metzeltin	0	0	0	0	
GACD	Gomphonema acidoclinatiforme	Metzeltin & Lange-Bertalot	0	0	0	0	
GACU*	Gomphonema acuminatum var. acuminatum	Ehrenberg	0	0	0	0	
GADC	Gomphonema acidoclinatum	Lange-Bertalot & Reichardt	1	1	1	1	
GAFF	Gomphonema affine	Kützing	0	0	0	0	
GAGU	Gomphonema angustius	E. Reichardt	0	0	0	0	
GAGV	Gomphonema angustivalva	E. Reichardt	0	0	0	0	
GANG	Gomphonema angustatum	(Kützing) Rabenhorst	0	0	1	1	
GANT	Gomphonema angustum	Agardh sensu Reichardt & Lange Bertalot	0	0	0	0	
GANT	Gomphonema angustum	Agardh	0	0	0	0	
GAUG	Gomphonema augur	Ehrenberg	0	0	0	0	
GAUR	Gomphonema auritum	A. Braun ex Kützing	0	0	0	0	
GBOB	Gomphonema bourbonense	E. Reichardt et Lange-Bertalot	1	1	1	1	
GBRE	Gomphonema brebissonii	Kützing	0	0	0	0	
GCAD	Gomphonema campodunense	E.Reichardt	0	0	1	0	
GCAP	Gomphonema capitatum	Ehrenberg	0	0	0	0	
GCLA	Gomphonema clavatum	Ehrenberg	0	0	0	1	
GCOR	Gomphonema coronatum	Ehrenberg	0	0	0	0	
GCUN	Gomphonema cuneolus	E. Reichardt	0	0	0	0	
GCUV	Gomphonema curvipedatum	H. Kobayasi ex Osada	0	0	0	0	
GCUW	Geissleria cummerowi	(L. Kalbe) Lange-Bertalot	0	0	0	0	
GELG	Gomphonema elegantissimum	Reichardt & Lange-Bertalot in Hofmann & al.	0	0	0	0	
GERI	Gomphoneis eriense	(Grunow) Skvortzow & Meyer	0	0	0	0	
GEXL	Gomphonema exilissimum	(Grun.) Lange-Bertalot & Reichardt	0	0	0	0	
GGDI	Gomphonema graciledictum	E.Reichardt	0	0	1	0	
GGRA	Gomphonema gracile	Ehrenberg	0	0	0	0	
GHEB	Gomphonema hebridense	Gregory	0	0	0	0	
GITA	Gomphonema italicum	Kützing	0	0	0	0	
GLAT	Gomphonema lateripunctatum	Reichardt & Lange-Bertalot	0	0	0	0	
GLGN	Gomphonema lagenula	Kützing	0	0	0	0	
GLOV	Gomphonella olivacea	NA	1	1	0	0	
GLTC	Gomphonema laticollum	Reichardt	0	0	0	0	
GMEX	Gomphonema mexicanum	Grunow	0	0	0	0	
GMIC*	Gomphonema micropus var. micropus	Kützing	0	0	0	0	
GMIN*	Gomphonema minutum f. minutum	(Ag.)Agardh	1	1	1	1	
GMIS	Gomphonema minusculum	Krasske	0	0	0	0	
GNLC	Gomphonella calcarea	(Cleve) R.Jahn & N.Abarca, comb. nov.	0	0	0	0	
GNVC	Gomphonema naviculoides	W. Smith	0	0	0	0	
GOCU	Gomphonema occultum	Reichardt & Lange-Bertalot	0	0	0	0	
GOLD	Gomphonema olivaceoides	Hustedt	0	0	0	0	
GPAN	Gomphocymbellopsis ancyli	(Cleve) Krammer	0	0	0	0	
GPAR*	Gomphonema parvulum var. parvulum f. parvulum	(Kützing) Kützing	1	1	1	1	
GPLI	Gomphosphenia lingulatiformis	(Lange-Bertalot & Reichardt) Lange-Bertalot	0	1	0	0	
GPSA	Gomphonema pseudoaugur	Lange-Bertalot	0	0	0	0	
GPUM	Gomphonema pumilum	(Grunow) Reichardt & Lange-Bertalot	1	1	1	1	
GRHB	Gomphonema rhombicum	M. Schmidt	0	0	0	0	
GRHB	Gomphonema rhombicum	Fricke	0	0	0	0	
GSBG	Gomphonema subangustatum	Lange-Bertalot Cavacini Tagliaventi & Alfinito	0	0	0	0	
GSCI	Gyrosigma sciotoense	(Sullivan et Wormley) Cleve	0	0	0	0	
GSCL	Gomphonema subclavatum	Grunow	1	1	1	1	
GSPP	Gomphonema saprophilum	(Lange-Bertalot & Reichardt) Abarca, R. Jahn, J. Zimmermann & Enke	0	1	1	1	
GTER	Gomphonema tergestinum	(Grunow in Van Heurck) Schmidt in Schmidt & al.	0	0	0	0	
GTNO	Gomphonema tenoccultum	Reichardt	0	0	0	0	
GTRU	Gomphonema truncatum	Ehrenberg	0	0	0	0	
GVIB	Gomphonema vibrio	Ehrenberg	0	0	0	0	
GVRD	Gomphonema varioreduncum	Jüttner, Ector, Reichardt, Van de Vijver & Cox	0	0	0	0	
GYAT	Gyrosigma attenuatum	(Kützing) Rabenhorst	0	0	0	0	
GYKU	Gyrosigma kuetzingii	(Grunow) Cleve	0	0	0	0	
HARC	Hannaea arcus	(Ehr.)Patrick	0	0	0	0	
HCAP	Hippodonta capitata	(Ehr.)Lange-Bert.Metzeltin & Witkowski	0	1	0	0	
HCOS	Hippodonta costulata	(Grunow)Lange-Bertalot Metzeltin & Witkowski	0	1	0	1	
HLMO	Halamphora montana	(Krasske) Levkov,	0	0	0	0	
HNEG	Hippodonta neglecta	Lange-Bertalot Metzeltin & Witkowski	0	0	0	0	
HOLI	Halamphora oligotraphenta	(Lange-Bertalot) Levkov	0	0	0	0	
HPDA	Hippodonta pseudacceptata	(Kobayasi) Lange-Bertalot Metzeltin & Witkowski	0	0	0	0	
HPEP	Humidophila perpusilla	(Grunow) Lowe, Kociolek, Johansen,Van de Vijver, Lange-Bertalot & Kopalová	0	0	0	0	
HSMA	Humidophila schmassmannii	(Hustedt) Buczkó et Wojtal	0	0	0	0	
HTHU	Halamphora thumensis	(A.Mayer) Levkov	0	0	0	0	
HUCO	Humidophila contenta	(Grunow) Lowe, Kociolek, Johansen, Van de Vijver, Lange-Bertalot & Kopalová	0	0	0	0	
HVEN	Halamphora veneta	(Kützing) Levkov,	0	0	0	0	
IDEL	Iconella delicatissima	(Lewis) Ruck & Nakov	0	0	0	0	
KALA	Karayevia laterostrata	(Hustedt) Bukhtiyarova	0	0	0	0	
KALA	Karayevia laterostrata	(Hustedt) Kingston	0	0	0	0	
KAPL	Karayevia ploenensis	(Hustedt) Bukhtiyarova	0	0	0	0	
KCLE*	Karayevia clevei var. clevei	(Grunow) Bukhtiyarova	0	0	0	0	
LBAL	Lindavia balatonis	(Pantocsek) Nakov, Guillory, Julius, Theriot & Alverson	0	0	0	0	
LGOP	Luticola goeppertiana	(Bleisch in Rabenhorst)D.G. Mann in Round Crawford & Mann	0	0	0	0	
LGOP	Luticola goeppertiana	(Bleisch) D.G.Mann ex J.Rarick, S.Wu, S.S.Lee & Edlund	0	0	0	0	
LHUN	Lemnicola hungarica	(Grunow) Round & Basson	0	0	0	0	
LPRA	Lindavia praetermissa	(Lund) Nakov, Guillory, Julius, Theriot & Alverson	0	0	0	0	
LRAD	Lindavia radiosa	(Grunow) De Toni & Forti	0	0	0	0	
MAAT*	Mayamaea atomus var. atomus	(Kützing) Lange-Bertalot	0	0	0	0	
MALC	Mayamaea alcimonica	(E. Reichardt) C.E. Wetzel, Barragán & Ector	0	0	0	0	
MCIR*	Meridion circulare var. circulare	(Greville) C.A. Agardh	0	0	0	0	
MING	Mayamaea ingenua	(Hustedt) Lange-Bertalot & Hofmann in Hofmann & al.	0	1	1	0	
MLAC	Mastogloia lacustris	(Grunow) van Heurck	0	0	0	0	
MPMI	Mayamaea permitis	(Hustedt) Bruder & Medlin	0	1	1	1	
MSMI	Mastogloia smithii	Thwaites	0	0	0	0	
MSTJ	Mastogloia sterijovskii	A. Pavlov. Jovanovska, C.E.Wetzel, Ector & Levkov	0	0	0	0	
MVAR	Melosira varians	Agardh	1	1	1	1	
NAAN	Navicula angusta	Grunow	0	0	0	0	
NACD	Nitzschia acidoclinata	Lange-Bertalot	0	0	0	0	
NACI	Nitzschia acicularis	Kützing) W.M.Smith	1	1	1	1	
NACU	Nitzschia acula	Hantzsch ex Cleve & Grunow	0	0	0	0	
NAGN	Nitzschia agnita	Hustedt	0	0	0	0	
NAGW	Nitzschia agnewii	Cholnoky	0	0	0	0	
NALP	Neidium alpinum	Hustedt	0	0	0	0	
NAMP*	Nitzschia amphibia f. amphibia	Grunow	1	1	1	1	
NANT	Navicula antonii	Lange-Bertalot	1	1	1	1	
NAPB	Nitzschia alpinobacillum	Lange-Bertalot	0	0	0	0	
NCAR	Navicula cari	Ehrenberg	0	0	0	0	
NCAT	Navicula catalanogermanica	Lange-Bertalot & Hofmann	0	0	0	0	
NCIN	Navicula cincta	(Ehr.) Ralfs in Pritchard	0	0	0	0	
NCLA	Nitzschia clausii	Hantzsch	0	0	0	0	
NCPL	Nitzschia capitellata	Hustedt in A. Schmidt & al.	1	1	1	1	
NCPR	Navicula capitatoradiata	Germain	1	1	1	1	
NCRY	Navicula cryptocephala	Kützing	0	0	1	0	
NCTE	Navicula cryptotenella	Lange-Bertalot	0	0	0	0	
NCTO	Navicula cryptotenelloides	Lange-Bertalot	0	1	0	0	
NCTT	Navicula cataracta-rheni	Lange-Bertalot	1	1	0	1	
NCTV	Navicula caterva	Hohn & Hellerman	0	1	0	0	
NDBF	Neidiomorpha binodeformis	Cantonati, Lange-Bertalot & Angeli	0	0	0	0	
NDIS*	Nitzschia dissipata subsp. dissipata	(Kützing) Grunow	0	1	0	0	
NDRA	Nitzschia draveillensis	Coste & Ricard	0	1	1	1	
NEDU	Neidium dubium	(Ehrenberg)Cleve	0	0	0	0	
NERI	Navicula erifuga	Lange-Bertalot in Krammer & Lange-Bertalot	0	0	0	0	
NEUT	Nitzschia eutinensis	Lange-Bertalot & Werum	0	0	0	0	
NEXI	Navicula exilis	Kützing	0	0	0	0	
NFIL*	Nitzschia filiformis var. filiformis	(W.M.Smith) Van Heurck	1	1	0	1	
NFON	Nitzschia fonticola	Grunow in Cleve et Möller	0	0	0	0	
NFSO	Nanofrustulum sopotensis	(Witkowski & Lange-Bert.) E.Morales, C.E.Wetzel & Ector, comb. nov.	0	0	0	1	
NFTR	Nanofrustulum trainori	(E.Morales) E.Morales, comb. nov.	1	1	0	1	
NGDU	Navigeia decussis	(Østrup) Bukhtiyarova	0	0	0	0	
NGER	Navicula germainii	Wallace	0	1	0	1	
NGES	Nitzschia gessneri	Hustedt	0	0	0	0	
NGHI	Navigeia hinziae	(Novais et Ector) Bukhtiyarova	0	0	0	0	
NGOT	Navicula gottlandica	Grunow in Van Heurck	0	0	0	0	
NGRE	Navicula gregaria	Donkin	0	0	0	1	
NHAN	Nitzschia hantzschiana	Rabenhorst	0	0	0	0	
NHEU	Nitzschia heufleriana	Grunow	0	0	0	0	
NHIN	Navicula hintzii	Lange-Bertalot	0	1	0	1	
NHMD	Navicula heimansioides	Lange-Bertalot	0	0	0	0	
NIAR	Nitzschia archibaldii	Lange-Bertalot	0	0	0	0	
NIBU	Nitzschia bulnheimiana	(Rabenhorst) H.L.Smith	0	0	0	0	
NIFQ	Nitzschia frequens	Hustedt	0	1	0	1	
NIFR*	Nitzschia frustulum var. frustulum	(Kützing) Grunow	1	1	1	1	
NIFT	Nitzschia fruticosa	Hustedt	0	0	0	0	
NIGR	Nitzschia gracilis	Hantzsch	1	1	1	1	
NILA	Nitzschia lacuum	Lange-Bertalot	0	0	0	0	
NIME	Nitzschia media	Hantzsch.	0	0	0	0	
NINC	Nitzschia inconspicua	Grunow	1	1	1	1	
NINT	Nitzschia intermedia	Hantzsch ex Cleve & Grunow	1	1	1	1	
NIOG	Nitzschia oligotraphenta	(Lange-Bertalot) Lange-Bertalot in Hofmann & al.	0	0	0	0	
NIPF	Nitzschia paleaeformis	Hustedt	0	0	0	0	
NIPM	Nitzschia perminuta	(Grunow) M.Peragallo	0	0	0	0	
NISO	Nitzschia solita	Hustedt	0	0	0	0	
NISU	Nitzschia subtilis	Grunow in Cleve et Grunow	0	0	0	0	
NIVA	Nitzschia valdestriata	Aleem & Hustedt	0	0	0	0	
NJOC	Navicula johncarterii	D.M.Williams	0	0	0	0	
NLAL	Nitzschia labella	Moser Lange-Bertalot & Metzeltin	0	0	0	0	
NLAN	Navicula lanceolata	(Agardh) Ehrenberg	0	0	0	0	
NLAN	Navicula lanceolata	(Agardh) Kützing	0	0	0	0	
NLIN*	Nitzschia linearis var. linearis	(Agardh) W.M.Smith	0	0	0	0	
NLTK	Navicula leistikowii	Lange-Bertalot	0	0	0	0	
NLUN	Navicula lundii	Reichardt	0	0	0	0	
NMCA	Navicula microcari	Lange-Bertalot	0	0	0	0	
NMEN*	Navicula menisculus var. menisculus	Schumann	0	0	0	0	
NMIC	Nitzschia microcephala	Grunow in Cleve & Moller	0	0	0	0	
NMOK	Navicula moskalii	Metzeltin, Witkowski & Lange-Bertalot	0	0	0	0	
NMTA	Navicula metareichardtiana	Lange-Bertalot & Kusber nom.nov.	0	1	0	1	
NNAN	Nitzschia nana	Grunow in Van Heurck	0	0	0	0	
NNOT	Navicula notha	Wallace	0	0	0	0	
NOBL	Navicula oblonga	Kützing	0	0	0	0	
NOLI	Navicula oligotraphenta	Lange-Bertalot & Hofmann	0	0	0	0	
NPAE	Nitzschia paleacea	(Grunow) Grunow in Van Heurck	1	1	1	1	
NPAL*	Nitzschia palea var. palea	(Kützing) W.Smith	1	1	0	1	
NPML	Nitzschia pumila	Hustedt	1	1	1	1	
NPRA	Navicula praeterita	Hustedt	0	0	0	0	
NPSL	Navicula pseudolanceolata	Lange-Bertalot	0	0	0	0	
NPUF	Nitzschia puriformis	Hlubikova et Ector	0	0	0	0	
NRAD	Navicula radiosa	Kützing	0	0	0	0	
NRCS	Navicula recens	(Lange-Bertalot) Lange-Bertalot	0	0	0	0	
NREC	Nitzschia recta	Hantzsch in Rabenhorst	0	0	0	0	
NRFA	Navicula radiosafallax	Lange-Bertalot	0	0	0	0	
NRHT	Navicula rhynchotella	Lange-Bertalot	0	0	0	0	
NRHY	Navicula rhynchocephala	Kützing	0	0	0	0	
NROS	Navicula rostellata	Kützing	0	0	0	0	
NSBL	Nitzschia sublinearis	Hustedt	0	0	0	0	
NSBN	Navicula subalpina	Reichardt	0	0	0	0	
NSIA	Navicula simulata	Manguin	1	1	1	1	
NSNM	Navicula sancti-naumii	Levkov, et Metzeltin	0	0	0	0	
NSOC	Nitzschia sociabilis	Hustedt	0	1	0	0	
NSOL	Nitzschia solgensis	Cleve-Euler	1	1	1	1	
NSTS	Nitzschia soratensis	Morales & Vis	1	1	0	1	
NSUA	Nitzschia subacicularis	Hustedt in A. Schmidt et al.	1	1	1	1	
NTAB	Nitzschia tabellaria	(Grun.) Grun. in Cl. & Grunow	0	1	0	0	
NTCX	Navicula trophicatrix	Lange-Bertalot	0	0	0	0	
NTPT	Navicula tripunctata	(O.F.Müller) Bory	1	1	1	1	
NTRV*	Navicula trivialis var. trivialis	Lange-Bertalot	0	0	0	0	
NUSA	Navicula upsaliensis	(Grunow) Peragallo	0	0	0	0	
NUVI	Nupela vitiosa	(Schimanski) Lange-Bertalot in Krammer & Lange-Bertalot	0	0	0	0	
NUWE	Nupela wellneri	(Lange-Bertalot) Lange-Bertalot	0	0	0	0	
NVDA*	Navicula vandamii var. vandamii	Schoeman & Archibald	0	1	0	0	
NVEN	Navicula veneta	Kützing	0	1	1	1	
NVGL	Navicula virginalis	Hustedt	0	0	0	0	
NVIR*	Navicula viridula var. viridula	(Kützing) Ehrenberg	0	0	0	0	
NVXP	Nitzschia vixpalea	Lange-Bertalot & Werum	0	0	0	0	
NWIL	Navicula wildii	Lange-Bertalot	0	0	0	0	
NXAS	Navicula associata	Lange-Bertalot	0	0	0	0	
NYCO	Nitzschia costei	Tudesque, Rimet & Ector	0	0	0	0	
NZAL	Nitzschia alpina	Hustedt	0	0	0	0	
NZRA	Nitzschia radicula	Hustedt	0	0	0	0	
NZSU	Nitzschia supralitorea	Lange-Bertalot	0	1	0	0	
PABV	Planothidium abbreviatum	(Reimer) Potapova	1	0	0	1	
PADE	Pantocsekiella delicatula	(Hustedt) K.T. Kiss et Ács	0	0	0	0	
PALT	Psammothidium altaicum	(Poretzky) Bukhtiyarova in Bukhtiyarova & Round	0	0	0	0	
PALV	Pseudostaurosira alvareziae	Cejudo-Figueras Morales & Ector	0	0	0	1	
PCIT	Playaensis citrus	(Krasske) E. Reichardt nov. comb.	0	0	0	0	
PCLD	Placoneis clementioides	(Hustedt) Cox	0	0	0	0	
PCLT	Placoneis clementis	(Grunow) Cox	0	0	0	0	
PCMS	Pantocsekiella comensis	(Grunow in Van Heurck) K.T. Kiss et Ács	0	0	0	0	
PCOS	Pantocsekiella costei	(Druart et F. Straub) K.T. Kiss et Ács	0	0	0	0	
PDAU	Planothidium daui	(Foged) Lange-Bertalot	0	1	0	0	
PDID	Psammothidium didymum	(Hustedt ) Bukhtiyarova et Round	0	0	0	0	
PDOP	Pseudostaurosira parasitoides	(Lange-Bertalot, Rol.Schmidt & Klee in Schmidt et al.) E.Morales, M.L.García & Maidana	0	0	0	0	
PDPC	Pseudostaurosiropsis connecticutensis	Morales	1	0	0	1	
PENA	Pennate diatom diatomée pennée non identifiée	in Kelly application TDI	0	0	0	0	
PFIB	Peronia fibula	(Brébisson ex Kützing)Ross	0	0	0	0	
PGRI	Psammothidium grischunum	(Wuthrich) Bukhtiyarova et Round	0	0	0	0	
PGRN	Planothidium granum	(Hohn & Hellerman) Lange-Bertalot	1	0	0	1	
PHEL	Psammothidium helveticum	(Hustedt) Bukhtiyarova et Round	0	0	0	0	
PKUE	Psammothidium kuelbsii	(Lange-Bertalot in L.-B. & K.) Bukhtiyarova et Round	0	0	0	0	
PLFR	Planothidium frequentissimum	(Lange-Bertalot) Lange-Bertalot	0	0	0	0	
PLFR	Planothidium frequentissimum	(Lange-Bertalot) Lange-Bertalot	0	0	0	0	
PLFR	Planothidium frequentissimum	(Lange-Bertalot) Lange-Bertalot	0	0	0	0	
PLHO	Platessa holsatica	(Hustedt) Lange-Bertalot	0	0	0	0	
PLHU	Platessa hustedtii	(Krasske) Lange-Bertalot	0	0	0	0	
PLJO	Platessa joursacense	(Héribaud) Chudaev in Chudaev. Gololobova et Kulikovskiy	0	0	0	0	
PLPM	Planothidium pumilum	B¹k & Lange-Bertalot	0	0	0	0	
PLVA	Psammothidium levanderi	(Hustedt) Bukhtiyarova	0	0	0	0	
PLVA	Psammothidium levanderi	(Hustedt) Czarnecki in Czarnecki et Edlund	0	0	0	0	
PLVU	Psammothidium lacus-vulcani	(Lange-Bert. et Kram.) Bukht. et Round	0	0	0	0	
PMNT	Planothidium minutissimum	(Krasske) Lange-Bertalot	0	0	0	0	
PMNT	Planothidium minutissimum	(Krasske) Morales	0	0	0	0	
PMOC	Pseudofallacia monoculata	(Hustedt) Liu Kociolek & Wang	0	0	0	0	
PMSC*	Pseudostaurosira microstriata var. microstriata	(Marciniak) Flower	0	0	0	0	
PMUL	Planothidium minusculum	(Hustedt) Witkowski, Kulikovskiy et Pliñski	0	0	0	0	
POBL	Platessa oblongella	(Østrup) C.E. Wetzel, Lange-Bertalot & Ector	0	0	0	0	
POCL	Pantocsekiella ocellata	(Pantocsek) K.T. Kiss et Ács	0	0	0	0	
POVA	Punctastriata ovalis	Williams & Round	0	0	0	0	
PPRS	Pseudostaurosira parasitica	(W.Smith) Morales	0	0	0	0	
PPSA	Placoneis pseudanglica	(Lange-Bertalot) Cox	0	1	0	0	
PRBU	Planothidium robustius	(Hustedt) Lange-Bertalot	0	0	0	0	
PROH	Planothidium rostratoholarcticum	Lange-Bertalot & B¹k	1	1	1	0	
PROS	Psammothidium rossii	(Hustedt) Bukhtiyarova et Round	0	0	0	0	
PRST	Planothidium rostratum	(Østrup) Lange-Bertalot	0	1	0	0	
PRST	Planothidium rostratum	(Østrup) Round & Bukhtiyarova	0	1	0	0	
PSBR	Pseudostaurosira brevistriata	(Grun.in Van Heurck) Williams & Round	0	0	0	0	
PSCA*	Pinnularia subcapitata var. subcapitata	Gregory	0	0	0	0	
PSCT	Psammothidium scoticum	(Flower & Jones) Bukhtiyarova et Round	0	0	0	0	
PSME	Pseudostaurosira medliniae	D.M.Williams & Morales	0	0	0	0	
PSPO	Pseudostaurosira polonica	(Witak & Lange-Bertalot) Morales et M.B. Edlund	0	0	0	0	
PSRE	Psammothidium rechtensis	(Leclercq) Lange-Bertalot	0	0	0	0	
PSSE	Pseudostaurosira elliptica	(Gasse) Jung & Medlin	0	0	0	0	
PSSE	Pseudostaurosira elliptica	(Schumann) Edlund, Morales & Spaulding	0	0	0	0	
PSYM	Placoneis symmetrica	(Hustedt) Lange-Bertalot	0	0	0	0	
PTCO	Platessa conspicua	(A.Mayer) Lange-Bertalot	0	0	0	0	
PTDE	Planothidium delicatulum	(Kütz.) Round & Bukhtiyarova	0	1	0	1	
PTDU	Planothidium dubium	(Grunow) Round & Bukhtiyarova	0	0	0	0	
PTLA	Planothidium lanceolatum	(Brébisson) Round et Bukhtiyarova	0	0	0	0	
PTLA	Planothidium lanceolatum	(Brébisson ex Kützing) Lange-Bertalot	0	0	0	0	
PTLA	Planothidium lanceolatum	(Brébisson in Kützing) Bukhtiyarova	0	0	0	0	
PTPU	Praestephanos triporus	(Genkal & G.V. Kuzmin)Tuji & J.-S. Ki	1	1	1	1	
PUDI	Punctastriata discoidea	Flower	0	0	0	0	
PULA	Punctastriata lancettula	(Schumann) Hamilton & Siver	0	0	0	0	
PUSB	Pseudostaurosira subconstricta	(Grunow) Kulikovskiy & Genkal ., stat. nov.	0	0	0	0	
PVEN	Psammothidium ventrale	(Krasske) Bukhtiyarova et Round	0	0	0	0	
PWUE	Pantocsekiella wuethrichiana	(Druart et F. Straub) K.T. Kiss et Ács	0	0	0	0	
PZIE	Platessa ziegleri	(Lange-Bertalot) Lange-Bertalot	0	0	0	0	
RABB	Rhoicosphenia abbreviata	(C.Agardh) Lange-Bertalot	1	0	1	1	
RFON	Reimeria fontinalis	Levkov, & Ector	0	0	0	0	
ROVA	Reimeria ovata	(Hustedt) Levkov, & Ector	0	0	0	0	
RPUS	Rossithidium pusillum	(Grunow) F.E.Round & Bukhtiyarova	0	0	0	0	
RSIN	Reimeria sinuata	(Gregory) Kociolek & Stoermer	0	0	0	0	
RUNI	Reimeria uniseriata	Sala Guerrero & Ferrario	0	0	0	0	
SACB	Sellaphora archibaldii	(J.C. Taylor et Lange–Bertalot) Ács, C.E. Wetzel et Ector .	0	0	0	0	
SANG	Surirella angusta	Kützing	0	0	0	0	
SARV	Sellaphora arvensis	(Hustedt) C.E. Wetzel et Ector	0	0	0	0	
SBND	Staurosira binodis	(Ehrenberg) Lange-Bertalot in Hofmann Werum et Lange-Bertalot	0	0	0	0	
SBND	Staurosira binodis	(Ehrenb.) Kulikovskiy & Genkal	0	0	0	0	
SBOH	Surirella bohemica	Maly	0	0	0	0	
SBRE*	Surirella brebissonii var. brebissonii	Krammer & Lange-Bertalot	0	0	0	0	
SCAN	Staurosirella canariensis	(Lange-Bertalot) E. Morales, Ector, Maidana & Grana .	0	0	0	1	
SCHK	Sellaphora chistiakovae	(Kulikovskiy et Lange-Bertalot) Wetzel, Ector Van De Vijver,Compère & D.G.Mann	0	0	0	0	
SCON	Staurosira construens	Ehrenberg	0	0	0	0	
SCPO	Sellaphora cosmopolitana	(Lange-Bertalot) C.E. Wetzel et Ector	0	0	0	0	
SCRA	Sellaphora crassulexigua	(Reichardt) Wetzel, Ector, Van De Vijver, Compère & D.G.Mann	1	1	1	1	
SCRM	Stauroneis charlesreimeri	Lange-Bertalot & Metzeltin	0	0	0	0	
SEAT	Sellaphora atomoides	Wetzel & Ector	0	0	0	0	
SEBA	Sellaphora bacillum	(Ehrenberg) D.G.Mann	0	0	0	0	
SECA	Sellaphora capitata	D.G. Mann & S.M. Mc Donald	0	0	0	0	
SELA	Sellaphora laevissima	(Kützing) D.G. Mann	0	0	0	0	
SELO	Sellaphora elorantana	(Lange-Bertalot) C.E. Wetzel	0	0	0	0	
SEUT	Sellaphora utermoehlii	(Hustedt) C.E. Wetzel et D.G. Mann	0	0	0	0	
SEXG	Stauroforma exiguiformis	(Lange-Bertalot) Flower Jones et Round	0	0	0	0	
SGRL	Stauroneis gracilior	Reichardt	0	0	0	0	
SGRL	Stauroneis gracilior	Reichardt in Van de Vijver & al.	0	0	0	0	
SHAN	Stephanodiscus hantzschii	Grunow in Cleve & Grunow	0	0	0	0	
SIDE	Simonsenia delognei	Lange-Bertalot	0	1	1	1	
SINM	Stauroforma inermis	Flower Jones et Round	1	1	0	1	
SKOS	Skabitschewskia œstrupii	(Cleve-Euler) Kulikovskiy & Lange-Bertalot	0	0	0	0	
SKPO	Skeletonema potamos	(Weber) Hasle	0	0	0	0	
SKRM	Staurosirella krammeri	E.A.Morales, C.Wetzel & Ector	0	0	0	0	
SLAC	Surirella lacrimula	English	0	0	0	0	
SLEP	Staurosirella leptostauron	(Ehr.) Williams & Round	0	0	0	0	
SLMU	Staurosirella mutabilis	(W. Smith) E. Morales & Van de Vijver	0	0	0	0	
SLPP	Staurosira lapponica	(Grunow) Lange-Bertalot	0	0	0	0	
SMTO	Sellaphora mutatoides	Lange-Bertalot & Metzeltin	0	0	0	0	
SNEO	Stephanodiscus neoastraea	Håkansson et Hickel	0	0	0	0	
SNIG	Sellaphora nigri	C.E. Wetzel et Ector . emend	1	1	1	1	
SNPI	Staurosirella neopinnata	E.A. Morales, C.E. Wetzel, E.Y. Haworth & L. Ector	0	0	0	0	
SODB	Staurosira oldenburgiana	(Hustedt)Lange-Bertalot	0	1	0	1	
SPCO	Staurosira pseudoconstruens	(Marciniak) Lange-Bertalot	0	0	0	0	
SPDV	Sellaphora pseudoarvensis	(Hustedt) C.E. Wetzel et Ector	0	0	0	1	
SPHO	Stauroneis phoenicenteron	(Nitzsch.) Ehrenberg	0	0	0	0	
SPIN	Staurosirella pinnata	(Ehrenberg) Williams&Round	0	0	0	0	
SPRG	Skabitschewskia peragalli	(Brun & Héribaud) Kulikovskiy & Lange-Bertalot	0	0	0	0	
SPSV	Sellaphora pseudoventralis	(Hustedt) Chudaev et Gololobova	0	0	0	0	
SPSV	Sellaphora pseudoventralis	(Hustedt) Wetzel, Ector Van De Vijver, Compère & D.G.Mann. Mann	0	0	0	0	
SPUP	Sellaphora pupula	(Kützing) Mereschkowksy	1	1	1	0	
SRAE	Sellaphora raederae	(Lange-Bertalot) C.E. Wetzel	0	0	0	0	
SRBU	Staurosira robusta	(Fusey) Lange-Bertalot	0	0	0	0	
SRHE	Sellaphora rhombelliptica	(Gerd Moser, Lange-Bertalot et Metzeltin) C.E. Wetzel et Ector	0	0	0	0	
SRMA	Staurosira martyi	(Héribaud) Lange-Bertalot	0	0	0	0	
SRTU	Sellaphora rotunda	(Hustedt) Wetzel, Ector Van De Vijver, Compère & D.G.Mann. Mann	0	0	0	0	
SSBG	Sellaphora schaumburgii	(Lange-Bertalot et G. Hofmann) C.E. Wetzel & Ector	0	0	0	0	
SSGE	Sellaphora saugerresii	(Desm.) C.E. Wetzel & D.G. Mann in Wetzel et al.	1	1	1	1	
SSMI	Stauroneis smithii	Grunow	0	0	0	0	
SSRT	Sellaphora subrotundata	(Hustedt) Wetzel, Ector Van De Vijver, Compère & D.G.Mann. Mann	0	0	0	0	
SSTM	Sellaphora stroemii	(Hustedt) Kobayasi in Mayama Idei Osada & Nagumo	0	0	0	0	
SSVE	Staurosira venter	(Ehrenberg) Cleve & Moeller	0	0	0	0	
SSVE	Staurosira venter	(Ehr.) H.Kobayasi	0	0	0	0	
SSVE	Staurosira venter	(Ehrenberg) Grunow in Pantocsek	0	0	0	0	
STLG	Staurosirella grunowii	(Pantocsek) E. Morales, Buczkó & Ector	0	0	0	0	
STMI	Stephanodiscus minutulus	(Kützing) Cleve & Moller	0	0	0	0	
STOV	Staurosirella ovata	Morales	0	0	0	0	
STSB	Staurosira berolinensis	(Lemm.)Kulikovskiy & Genkal	0	0	0	0	
STSB	Staurosira berolinensis	(Lemm.) Lange-Bertalot	0	0	0	0	
STSE	Stauroneis separanda	Lange-Bertalot & Werum	0	0	0	0	
STTU	Stephanodiscus tenuis	Hustedt	0	0	0	0	
SULI	Surirella librile	(Ehrenberg Ehrenberg	0	0	0	0	
SUUN	Surirella undulata	(Ehrenberg) Ehrenberg	0	0	0	0	
SVTB	Sellaphora vitabundicta	E. Reichardt nov. spec.	0	0	0	0	
SVTL	Sellaphora ventraloides	(Hustedt) Falasco & Ector	0	0	0	0	
TANG	Tryblionella angustata	W.M. Smith	0	0	0	0	
TATU	Tryblionella angustatula	(Lange-Bertalot) Cantonati & Lange-Bertalot in Kusber et al. .	0	1	0	1	
TBNO	Tryblionella brunoi	(Lange-Bertalot) Cantonati et Lange-Bertalot in Kusber et al.	0	0	0	0	
TCAL	Tryblionella calida	(Grunow in Cl. & Grun.) D.G. Mann in Round Crawford & Mann	0	0	0	0	
TFAS	Tabularia fasciculata	(Agardh)Williams et Round	0	0	0	0	
TFEN	Tabellaria fenestrata	(Lyngbye) Kützing	0	0	0	0	
TFLO	Tabellaria flocculosa	(Roth) Kützing	0	0	0	0	
TKUE	Tryblionella kuetzingii	Alvarez-Blanco & S.Blanco	0	0	0	0	
TLEV	Tryblionella levidensis	Wm. Smith	0	0	0	0	
TVEN	Tabellaria ventricosa	Kützing	0	0	0	0	
UACU	Ulnaria acus	(Kützing) Aboal	1	1	1	1	
UBIC	Ulnaria biceps	(Kützing) Compère	0	0	0	0	
UDEL	Ulnaria delicatissima	(W.Smith) Aboal & Silva	0	0	0	0	
UDEL	Ulnaria delicatissima	(W.Smith) Aboal	0	0	0	0	
UGRU	Ulnaria grunowii	(Lange-Bertalot et Ulrich) Cantonati et Lange-Bertalot in Kusber & al.	0	0	0	0	
UULN	Ulnaria ulna	(Nitzsch) Compère	1	0	1	1	
VUCO	Diatomées non identifiées vc	non identifiées vue connectives	0	0	0	0	
ZZZZ	Genre non identifie	NA	0	0	0	0	
