# Supplementary material for: A new diatom-based multimetric index to assess lake ecological status
Source: Environ Monit Assess. 2023 Sep 13;195(10):1202. doi: 10.1007/s10661-023-11855-w (PMC10499699; doi:10.1007/s10661-023-11855-w)
Supplement: Supplementary file 2 — Supplementary file2 (RTF 53 KB) [file 10661_2023_11855_MOESM2_ESM.rtf]

variable	SESnorRef	
BOD5	0.9358017	
MES	0.9081657	
NKJ	0.9138570	
NO2	0.8865007	
NO3	0.7155957	
PO4	0.9088790	
Pt	0.8880905	
cond__	0.7861347	
O2_dissous__	0.9069739	
Sat O2	0.9519387	
