# Supplementary material for: A new diatom-based multimetric index to assess lake ecological status
Source: Environ Monit Assess. 2023 Sep 13;195(10):1202. doi: 10.1007/s10661-023-11855-w (PMC10499699; doi:10.1007/s10661-023-11855-w)
Supplement: Supplementary file 3 — Supplementary file3 (RTF 61 KB) [file 10661_2023_11855_MOESM3_ESM.rtf]

variable	Min	Max	
BOD5	-4.248347	0.900000	
MES	-4.158029	1.000000	
NKJ	-4.804299	1.000000	
NO2	-5.388889	1.441964	
NO3	-4.191932	2.234127	
PO4	-5.271100	1.000000	
Pt	-3.559219	1.173913	
cond__	-5.280570	1.964286	
O2_dissous__	-7.668478	1.200000	
Sat O2	-12.871176	1.000000	
