# Supplementary material for: A new diatom-based multimetric index to assess lake ecological status
Source: Environ Monit Assess. 2023 Sep 13;195(10):1202. doi: 10.1007/s10661-023-11855-w (PMC10499699; doi:10.1007/s10661-023-11855-w)
Supplement: Supplementary file 4 — Supplementary file4 (RTF 47 KB) [file 10661_2023_11855_MOESM4_ESM.rtf]

variable	type	substrate	Mgroup	SDgroup	
Cond	LA	mineral	0.98	0.02	
Cond	MA	mineral	0.87	0.16	
Cond	HA	mineral	0.55	0.22	
Cond	LA	macrophyte	0.99	0.01	
Cond	MA	macrophyte	0.95	0.06	
Cond	HA	macrophyte	0.62	0.21	
BOD5	LA	mineral	0.98	0.03	
BOD5	MA	mineral	0.82	0.20	
BOD5	HA	mineral	0.88	0.18	
BOD5	LA	macrophyte	0.99	0.02	
BOD5	MA	macrophyte	0.87	0.19	
BOD5	HA	macrophyte	0.85	0.22	
MES	LA	mineral	0.96	0.04	
MES	MA	mineral	0.79	0.21	
MES	HA	mineral	0.74	0.27	
MES	LA	macrophyte	0.98	0.02	
MES	MA	macrophyte	0.90	0.19	
MES	HA	macrophyte	0.82	0.24	
NKJ	LA	mineral	0.97	0.03	
NKJ	MA	mineral	0.87	0.20	
NKJ	HA	mineral	0.90	0.16	
NKJ	LA	macrophyte	0.98	0.02	
NKJ	MA	macrophyte	0.91	0.18	
NKJ	HA	macrophyte	0.87	0.21	
NO2	LA	mineral	0.96	0.06	
NO2	MA	mineral	0.83	0.15	
NO2	HA	mineral	0.75	0.17	
NO2	LA	macrophyte	0.98	0.02	
NO2	MA	macrophyte	0.84	0.20	
NO2	HA	macrophyte	0.77	0.19	
NO3	LA	mineral	0.89	0.11	
NO3	MA	mineral	0.85	0.16	
NO3	HA	mineral	0.58	0.18	
NO3	LA	macrophyte	0.92	0.08	
NO3	MA	macrophyte	0.90	0.13	
NO3	HA	macrophyte	0.68	0.16	
O2	LA	mineral	0.94	0.05	
O2	MA	mineral	0.94	0.08	
O2	HA	mineral	0.97	0.06	
O2	LA	macrophyte	0.96	0.04	
O2	MA	macrophyte	0.94	0.12	
O2	HA	macrophyte	0.97	0.08	
PO4	LA	mineral	0.97	0.03	
PO4	MA	mineral	0.84	0.21	
PO4	HA	mineral	0.78	0.25	
PO4	LA	macrophyte	0.98	0.02	
PO4	MA	macrophyte	0.94	0.14	
PO4	HA	macrophyte	0.84	0.23	
Pt	LA	mineral	0.93	0.06	
Pt	MA	mineral	0.73	0.23	
Pt	HA	mineral	0.79	0.25	
Pt	LA	macrophyte	0.98	0.02	
Pt	MA	macrophyte	0.83	0.24	
Pt	HA	macrophyte	0.85	0.21	
%O2	LA	mineral	0.99	0.01	
%O2	MA	mineral	0.99	0.03	
%O2	HA	mineral	1.00	0.01	
%O2	LA	macrophyte	1.00	0.01	
%O2	MA	macrophyte	0.97	0.07	
%O2	HA	macrophyte	0.99	0.03	
